# Autoimmune Thyroid Disease in Rheumatoid Arthritis: A Global Perspective

**DOI:** 10.1155/2012/864907

**Published:** 2012-11-18

**Authors:** Jorge Cárdenas Roldán, Jenny Amaya-Amaya, Juan Castellanos-de la Hoz, Juliana Giraldo-Villamil, Gladys Montoya-Ortiz, Paola Cruz-Tapias, Adriana Rojas-Villarraga, Rubén D. Mantilla, Juan-Manuel Anaya

**Affiliations:** ^1^Center for Autoimmune Diseases Research (CREA), School of Medicine and Health Sciences, Universidad del Rosario, Bogota, Colombia; ^2^Doctoral Program in Biomedical Sciences, Universidad del Rosario, Bogota, Colombia

## Abstract

*Objective*. To determine the prevalence and impact of autoimmune thyroid disease (AITD) in patients with rheumatoid arthritis (RA). *Methods*. Eight-hundred patients were included. The association between AITD and RA was analyzed was analyzed by bivariate and multivariate analysis. In addition, a literature review was done focusing on geographical variations. *Results*. In our cohort the prevalence of AITD was 9.8% while the presence of antibodies was 37.8% for antithyroperoxidase enzyme (TPOAb) and 20.8% for antithyroglobulin protein (TgAb). The presence of type 2 diabetes, thrombosis, abnormal body mass index, and a high educational level was positively associated with AITD. The literature review disclosed a geographical variation of AITD in RA ranging from 0.5% to 27%. Autoantibody prevalence ranges from 6% to 31% for TgAb, 5% to 37% for TPOAb, and from 11.4% to 32% for the presence of either of the two. *Conclusion*. AITD is not uncommon in RA and should be systematically assessed since it is a risk factor for developing diabetes and cardiovascular disease. These results may help to further study the common mechanisms of autoimmune diseases, to improve patients' outcome, and to define public health policies. An international consensus to accurately diagnose AITD is warranted.

## 1. Introduction

Autoimmune thyroid disease (AITD) is a term used to bring together a group of pathologies that has thyroid dysfunction and an autoimmune response against this endocrine organ as its hallmark [1, 2]. However, being a group of autoimmune diseases (ADs) clustered together, the clinical presentation varies among these diseases; it can be divided into those that cause hypothyroidism, hyperthyroidism, or both [3].

As organ specific ADs, this group of pathologies exhibits an autoantibody profile that may be composed of (1) antibodies directed against the thyroperoxidase enzyme (TPOAb), (2) antibodies directed against thyroglobulin protein (TgAb), and (3) antibodies directed against thyrotropin receptor (TSHrAb). In this last case, the antibodies can either block or enhance the receptor's activity. Furthermore, there is a T or B lymphocytic response that prevails and, ultimately, this will define the pathology that becomes manifest. Generally, T lymphocytes are the main cell type infiltrating the gland in Hashimoto's thyroiditis while a B response predominates and determines the presence of Grave's disease [3].

In general terms, those diseases where the clinical presentation is mainly a hypothyroid state include Hashimoto's thyroiditis. As originally described by Akaru Hashimoto in 1912 goiter was associated with this disease though today it may or may not be. The other disease is atrophic thyroiditis which is found with hypothyroidism in the absence of goiter. Conversely, Grave's disease, described by Robert Graves in 1835, is manifested by a hyperthyroid state that can be associated with diffuse goiter and sometimes with exophthalmos. Postpartum thyroiditis occurs in the first postpartum year and it may start with a hyperthyroid state and end with a hypothyroid state that can be transient or permanent [4].

The prevalence of AITD in the general population varies between countries. A prevalence has been described of 5 to 15% in women and 1–5% in men [5]. The prevalence of thyroid autoantibodies has also been described. Hollowell et al. [6] described a prevalence of 13% for TPOAb and 11.5% for TgAb among the general population. This prevalence rises in spontaneously hypothyroid patients [7]. In other words, AITD can be regarded as the most common autoimmune endocrine disease. 

Rheumatoid arthritis (RA), in turn, is a chronic, complex, and heterogeneous AD, in which there is a response directed towards the diarthrodial joints producing symmetric polyarthritis with progressive damage to the joints, bone destruction, and extra-articular manifestations (EAMs) such as cutaneous nodules, lung involvement, cardiovascular disease (CVD), episcleritis, and so forth. All of these lead to disability [8, 9], an increase in comorbidities [10], and premature mortality. Thus, the autoimmune compromise is systemic as opposed to AITD which is organ specific [11]. 

RA is the most common inflammatory arthropathy worldwide. The disease is three times more frequent in women than men with a prevalence of 0.5–1.0% in industrializedcountries [12, 13] and less than 0.5% in Latin America [12, 14]. This region has a high admixture of cultures and ethnicities and thus RA genotypes and phenotypes differ between and within countries [15]. However, the prevalence rises with age and is highest in women older than 65 years [11]. The annual incidence is highly variable (12 to 1,200 per 100,000 population) [16] and is dependent on a variety of factors including gender, environmental (e.g., smoking [17], infectious diseases [18, 19]), ethnicity, and age [16]. With the exception of certain native populations, RA affects all populations worldwide. These variations are indicative of different genetic risks and hormonal exposures [20].

For several decades an increased occurrence of thyroid disorders in patients suffering from RA has been documented—both autoimmune and nonautoimmune in nature [21–24]. In addition, [25] rheumatologic and nonrheumatologic manifestations of AITD have been described. Within these manifestations, it is noteworthy that the most common symptoms are polyarthralgia and unclassified arthritis, which are the main features of RA. 

 ADs share similar mechanisms [12, 26–28]. In clinical practice some conditions support these commonalities. One of these corresponds to polyautoimmunity, which is defined as the presence of more than one AD in a single patient [29]. The multiple autoimmune syndrome (MAS), a form of polyautoimmunity, corresponds to the coexistence of three or more well-defined ADs [30]. The importance of these terms is due to the fact that patients with polyautoimmunity or MAS may have a modified disease course (with a worse prognosis or a better one) and a modified clinical presentation. Moreover, first degree relatives (FDR) of these patients are at increased risk of developing an AD [31]. Several studies have consistently mentioned association and clustering between ADs [32, 33].

Genetic background is, therefore, an important aspect in autoimmunity. Genetic risk factors shared among diseases have been described and AITD and RA are no exception [25, 34, 35]. Nevertheless, the etiology of ADs is complex in nature, which means that genetic, epigenetic, and environmental factors are responsible for the occurrence of these diseases. For certain ADs, genetic factors have been consistently found to be more important than environmental factors and vice versa [1, 2, 36, 37]. 

In AITD, numerous genes have been found to confer risk for the disease including HLA gene complex, CD40, CTLA4, PTPN22, TSH receptor gene, and thyroglobulin gene [2, 38–40]. While the term AITD lumps Graves' disease and Hashimoto's thyroiditis together, in the case of the former, genetic factors appear to be more important whereas the reverse is true in the latter [1, 2, 36, 37]. CD40, CTLA4, and PTPN22 genes as well as the HLA gene complex have also been implicated in the pathogenesis of RA [41]. In addition, shared environmental factors such as smoking [17] have been implicated in numerous studies as risk factors for AITD and for RA [2, 36]. 

Although AITD and RA share common physiopathological mechanisms, the connection between AITD and RA is a topic with no definite results so far. In Latin America and other regions, this association has not been thoroughly explored. It is important to establish if the presence of AITD in RA is linked with EAMs including CVD and the presence of a worse prognosis for RA (e.g., presenting erosions) [42, 43]. As a center for autoimmune research established in Latin America, we are mainly interested in unraveling the association between these diseases, to look for information from our region and to establish a solid base on which future research in this area may hold its ground. 

The purposes of the study are (1) to determine the prevalence of AITD within an RA cohort of Colombian patients and determine the differences between these two groups regarding the prognostic features of RA as well as (2) to analyze the current information concerning the prevalence of AITD in RA patients and to evaluate any deviations on RA course due to AITD presence.

## 2. Patients and Methods

### 2.1. Study Population

This was a cross-sectional, analytical study in which 800 consecutive Colombian patients with RA were included. The subjects were being seen at the Center for Autoimmune Diseases Research (CREA) at the Universidad del Rosario in Bogota and Medellin between February 1996 and April 2012. All of them fulfilled the 1987 American College of Rheumatology classification criteria [44] and had AITD status investigated. The study was conducted in compliance with Act 008430/1993 by the Ministry of Health of the Republic of Colombia. The institutional review board of the Universidad del Rosario approved the study design.

Each patient was evaluated by a rheumatologist. The information on patient sociodemographic and cumulative clinical and laboratory data was obtained by interview, physical examination, and chart review. A household description was obtained by questionnaire and a clinical evaluation of the affected family members was done using the same methodology as above. All data were collected in an electronic and secure database. 

The sociodemographic variables included current age, age at RA onset, disease duration, educational status, socioeconomic status (SES), current occupational status, smoking habits, coffee consumption, and physical activity. The following are the definitions of these variables: age at onset: age at which patients began to suffer from pain, typical morning stiffness (more than 1 hour), and symmetrical inflammation of hand and/or foot joints; disease duration: difference between age at onset and the date of first participation in the study. It was dichotomized as having either more or less than 10 years of disease as our group had previously reported this to be a risk factor for CVD [45]. Educational level was recorded as years of education; the cohort was split into two groups with one group including those with less than 9 years of education (including preschool, primary, and the first 2-3 years of high school) and the other more than 9 years of education. This breakdown was based on the General Law of Education in Colombia [46, 47]. SES was categorized on the basis of national legislation and was divided into high status (3 to 6) and low status (1 and 2). For occupational status we focused on establishing if the patient worked on household duties exclusively.

Regarding clinical variables, we evaluated polyautoimmunity, MAS, familial autoimmunity, erosions, comorbidities, EAMs, systolic and diastolic blood pressure, body mass index (BMI), and waist circumference. The following are the definitions of these variables: polyautoimmunity and MAS as stated above. However, we evaluated polyautoimmunity as a variable without taking into account the presence of AITD. Familial autoimmunity was defined as the presence of any diagnosed AD in another FDR of the proband. We evaluated 6 ADs on the basis of international criteria namely: systemic lupus erythematosus (SLE), AITD, Sjögren's syndrome (SS), antiphospholipid syndrome (APS), psoriasis (PSO), and vitiligo (VIT) [48]. It is important to note that there are no international criteria for the diagnosis of AITD. These cases were classified on the basis of an abnormal thyrotropin (TSH) test, or history of thyroid hormone therapy, and the presence of either TPOAb or TgAb. 

Erosions were defined as having at least one point on the Sharp/van der Heijde classification [49]. EAMs was defined as the presence of at least one of the following: skin ulcerations, nodules, episcleritis, vasculitis, neuropathy, pleural effusion, pulmonary hypertension or embolism, and CVD. The latter was categorized as positive if any of the following variables were present: hypertension (defined as having blood pressure >140/90 mm Hg or using antihypertensive medication) [50], coronary artery disease, occlusive arterial disease, carotid disease, or thrombosis.

The patients were asked about the presence of diabetes mellitus, defined as having a fasting plasma glucose level >7 mmol/L (126 mg/dL) or taking antidiabetic medication at the time of the assessment [51]. Diagnosis of dyslipidemia was given if patient had hypercholesterolemia, defined as taking lipid-lowering medication or having a fasting plasma total cholesterol >200 mg/dL, HDL <40 mg/dL, hypertriglyceridemia >150 mg/dL, or LDL cholesterol >100 mg/dL [52, 53]. Anemia was diagnosed if current hemoglobin was <12 g/dL, gastritis only if evidenced by esophagogastroduodenoscopy, periodontal disease was self-reported, and renal disease if serum creatinine measurement had values above 1.2 mg/dL. 

Systolic and diastolic blood pressures were measured twice with at least 15 minutes between measurements and the average was recorded. A BMI ≥25 kg/m^2^ (overweight and obesity) was considered abnormal [54]. Abnormal values of waist circumference (>102 cm for men, >88 cm for women) and waist-to-hip ratio (WHR > 0.9 for men, >0.85 for women) were considered indicators of abdominal obesity. Waist circumference was measured around the narrowest point between ribs and hips after exhaling and viewed from the front. Hip circumference was measured at the point of maximum extension of the buttocks when viewed from the side [55]. Abnormal WHR values are consistent with National Cholesterol Education Program Adult Treatment Panel III and World Health Organization definitions [56]. 

Medical treatment included the current or past use of methotrexate and other disease modifying antirheumatic drugs (DMARDs) such as sulfasalazine, D-penicillamine, azathioprine, cyclosporine, gold salts and leflunomide, steroid therapy, antimalarials (cloroquine, hydroxychloroquine), and biologic therapy (rituximab, infliximab, etanercept, abatacept, adalimumab, tocilizumab). Patients and their past medical records were evaluated for the current or past use of aspirin or hormone replacement therapy as well.

Relevant laboratory variables were also registered including erythrocyte sedimentation rate (ESR), hemoglobin levels, white blood cell count, platelet count, and serum high sensitive C-reactive protein (CRP) levels. Autoantibodies such as rheumatoid factor (RF) and anticyclic citrullinated peptide (anti-CCP), TPOAb, and TgAb antibodies were taken from the patient's clinical record. They were measured with enzyme-linked immunosorbent assay (QUANTA-Lite, INOVA, San Diego, CA, USA) following the manufacturer's protocol. Antibodies directed against either TSH receptor or thyroid hormones (THAb) were not assessed in the current study.

### 2.2. Statistical Analysis

First, univariate analysis was done on all members of this new cohort. Categorical variables were analyzed by frequencies. Kolmogorov-Smirnov normality test was done to evaluate normality for quantitative, continuous variables. Parametric data are expressed as mean and standard deviation (SD), and nonparametric data are described as median and interquartile range (IQR).

Second, bivariate analyses done in search of the association between different characteristics of RA and AITD were verified using chi-square test or Fisher's exact test when the factors were dichotomous. Parametric values were analyzed by Student's *t*-test. Nonparametric values were analyzed by Mann-Whitney *U*-test. A *P* value of less than 0.05 was considered significant.

A multivariate binomial logistic regression model was fit with AITD as the dependent variable. As independent variables, the model included those that were significantly associated in the bivariate analyses and those that were biologically and clinically plausible for this relationship. The adequacy of logistic models was assessed using the Hosmer-Lemeshow goodness-of-fit test. The Nagelkerke *R* ^2^ (i.e., pseudo-*R* ^2^) was used to estimate the percentage of variance explained by the model. Adjusted odds ratios (AORs) were calculated with 95% confidence intervals (CIs). Statistical analyses were done by using the Statistical Package for the Social Sciences (SPSS, v.20, Chicago, IL).

### 2.3. Literature Search

We did a literature review with reference to polyautoimmunity between RA and AITD. The search was done using the following databases: PubMed, SciELO, EMBASE, Virtual Health Library (BIREME and LILACS), and Google Scholar.

Limits regarding language, age (all adults), and humans were taken into account. No limits regarding publication date was used. The following Medical Subject Headings (MeSH terms) were used: “Thyroiditis, Autoimmune” OR “Graves Disease” AND “Arthritis, Rheumatoid”. In addition, each MeSH term was translated into DeCS (Health Sciences Descriptors), a tool that makes it possible to navigate between records and sources of information through controlled concepts organized in Spanish and English. This was done in order to search SciELO, BIREME, and Virtual Health Library databses. The DeCs terms and key words used were “artritis reumatoide” AND (“tiroiditis autoinmune” OR “enfermedad de graves”).

The inclusion criteria were the following: only articles that used accepted classification criteria for RA had a definite diagnosis of AITD (presence of antithyroid antibodies and thyroid dysfunction), and that included RA as well as AITD. They were divided based on prevalence of AITD, prevalence of antithyroid antibodies, radiographic progression, and extra-articular manifestations. Articles were excluded if they were animal models, dealt with juvenile rheumatoid arthritis, or with other autoimmune diseases other than RA or AITD. 

Those references from the articles that seemed to be relevant for our review were hand searched. Titles and abstracts were reviewed by two independent reviewers in search of eligible studies. 

## 3. Results

### 3.1. Colombian Cohort

There were 81.3% women and we found that the prevalence of AITD was 9.8%. The presence of antibodies was 37.8% for TPOAb and 20.8% for TgAb. Characteristics of the cohort are illustrated in Table 1. Due to the nature of this study (i.e., cross-sectional) and the cohort beginning date (i.e., 1996) there is a proportion of patients in whom not all the data were assessed. Health assessment questionnaire (HAQ) and disease activity score (DAS28) were calculated on study entry date, but were not taken into account in the analyses due to their variability over time. 

In the bivariate analysis, significant differences among women, educational level, abnormal BMI, diabetes, thrombosis, hypercholesterolemia, presence of RF, and use of methotrexate were observed. Tables 2 and 3 show the relationships explored in the study population. 

Table 4 depicts the multiple logistic regression analysis. Adjusted for gender and RA duration, the presence of diabetes, thrombosis, and abnormal BMI were positively associated in patients with polyautoimmunity (i.e., between RA and AITD). We found that there is a lower AITD frequency in the lowest educational level than in the highest one. This is also true when antimalarials are used (Table 4). 

### 3.2. Literature Search Results

The searches in Medline, EMBASE, LILACS, and BIREME brought up 788 articles. Forty-nine were selected for further analysis based on their title and abstract. Using information from references, other studies that met the selection criteria were chosen. The articles were divided by measured outcomes that were considered relevant: radiographic progression, genetic analysis, prevalence of AITD, and prevalence of TPOAb or TgAb.

#### 3.2.1. Prevalence of AITD (Figure 1)

Seventeen studies identified RA as index disease and determined AITD prevalence in this group [23, 57–72]. The prevalence in RA cases ranged from 0.5% in Morocco [58] to 27% in Slovakia [71]. Within the studies analyzed, 10 studies were from Europe [23, 63–71] with prevalence ranging from 1% in Germany [65] to 27% in Slovakia [71]. Four studies were from the North American region [59–62] where prevalence ranged from 2.1% [61] to 9.8% [60]. Only two studies were from Africa were retrieved [57, 58] and one from the Middle East [72]. The search did not result in any article about Latin American or Asian populations.  Table 5 gives a detailed view of the data. 

#### 3.2.2. Prevalence of Autoantibodies (Figure 1)

Twenty studies reported the prevalence of autoantibodies against thyroid antigens [23, 57, 70, 73–89]. The prevalence for TgAb ranged from 5% in men from the UK [88] and 6% regardless of gender in Egypt [57] to 31% in RA patients from Japan [79]; the prevalence for TPOAb was within the range of 5% in Egypt [74] to 37% in Italy [80]. This search included 2 studies from Brazil [76, 77] and one from Argentina [75] which were the only countries from Latin America that had published literature on this topic. Some studies did not discriminate the frequency of each autoantibody [23, 76]. Ruggeri et al. [81] show the assessment of THAb at three points in time. Further information can be obtained from Table 6. 

#### 3.2.3. Extra-Articular Manifestations

In our search CVD was the sole EAMs found. Articles by McCoy et al. [62] and Raterman et al. [21] agreed that the presence of hypothyroidism, including Hashimoto's thyroiditis, is a risk factor for CVD in patients with RA. McCoy and colleagues found a hazard ratio of 2.7 (95% CI: 1.1–6.3) [62].

#### 3.2.4. RA Severity

One full text article and three abstracts were located that dealt with this topic. 

## 4. Discussion

In our cohort the prevalence of AITD was 9.8% while the presence of antibodies was 37.8% for TPOAb and 20.8% for TgAb. Type 2 diabetes (AOR: 13.61; 95% CI: 1.61–114.96; *P* = 0.016), thrombosis (AOR: 24.4; 95% CI: 2.72–218.42; *P* = 0.004), and abnormal BMI (AOR: 4.22; 95% CI: 1.19–14.93; *P* = 0.025) were positively associated in patients with polyautoimmunity (i.e., RA and AITD) while the lowest educational level (AOR: 0.16; 95% CI: 0.03–0.88; *P* = 0.036) as well as the use of antimalarials (AOR: 0.10; 95% CI: 0.18–0.57; *P* = 0.01) were negatively associated with this coexistence. 

There is a worldwide prevalence of AITD in RA that varies considerably, ranging from 0.5 % in Morocco [58] to 27% in Slovakia [71]. Thyroid-specific antibody prevalence ranges from 6 to 31% for TgAb [57, 79], 5 to 37% for TPOAb [74, 80], and from 10.4 to 32% for the presence of either of the two [23, 76]. This high prevalence variability may be explained by certain factors. Firstly there are difficulties on diagnosing AITD because it relies on the fact that there must be a diagnosis of thyroid dysfunction *a priori*. However there has been much of a debate regarding how to define hypothyroidism or hyperthyroidism; the normal reference range is not universally accepted and thus authors and clinicians worldwide accept different normal ranges. 

The debate is more intense when establishing the normal upper limit for TSH values; several authors have addressed this issue but there has been no consensus [90–96]. Wartofsky and Dickey [91], and the Wickham cohort propose a TSH range around 2.5 IU/mL [97] while Surks et al. [94] and the American Academy of Clinical Endocrinologists (AACE) [98] support a TSH upper limit of 5 IU/ml. Jensen et al. and Hamilton et al. [90, 95] found a normal upper TSH level of 4.1 IU/ml, which is more clinically acceptable in order for initiating therapy. Secondly, the TSH assay methods have changed with time, improving its diagnostic accuracy [96]. This may hold true for other assays. Older studies may have not detected low levels of a given laboratory value and thus report a false negative result. This could explain the results of Ruggeri et al. [81] in which the prevalence of THAb are increasing with time. 

A third explanation involves iodine intake. It is well known that iodine has a particular property of inducing autoimmune response against the thyroid [99–101]. Epidemiological studies support this statement as they have found an increasing incidence of AITD, particularly Hashimoto's thyroiditis, with increasing iodine intake (0.2% for low, 1% for normal, and 1.3% for high intake) [48]. Besides, a rise in Hashimoto's thyroiditis prevalence was encountered after adjustment of iodine supplementation [102]. Given this, and accepting the fact that iodine supplementation/intake is not evenly distributed among countries [103], it is plausible to think that this may also contribute to the heterogeneous prevalence of AITD in RA found around the globe. 

When polyautoimmunity was assessed without taking into account AITD, a prevalence of 5% was found, which is relatively high. This is linked to a positive association between Ro and La antibodies and AITD. Both of these findings are supported by the “Autoimmune Tautology” [27]. 

 Surprisingly, the association between AITD and EAMs did not become apparent in the literature search nor in our cohort. Although CVD is linked to the presence of EAMs [104], an increased cardiovascular risk is observed within this subset of patients with an OR of 3.1 in the bivariate analysis and an AOR of 24 when adjusted for potential confounders and variables of clinical interest. This is the reason CVD is considered an EAM and a major predictor of poor prognosis [16] and increased RA medical costs [105]. 

The aforementioned relationship found is supported by other studies. McCoy et al. [62] found that Hashimoto's disease had an HR of 2.1 (95% CI: 1.2–3.8) for CVD in patients with RA in a retrospective cohort. By perpetuating an inflammatory state RA is also considered as a novel risk factor for CVD. This has been shown in a large number of reports [22, 106, 107] and was also demonstrated in our cohort previously [45, 104].

Furthermore, higher ESR, CRP, and TNF-*α* titers, the occurrence of RA vasculitis, and RA lung disease emerged as strong disease-specific predictors of cardiovascular mortality. This also holds even after accounting for demographics, traditional cardiovascular risk factors such as diabetes, sedentary lifestyle, obesity, smoking, and relevant comorbidities [108]. It has been proposed that an altered lipid profile is responsible for excess of CVD in patients with AITD [109]. However, Taddei et al. [110] in a case-control setting compared patients with subclinical hypothyroidism and autoimmune thyroiditis versus controls. They found that low grade systemic inflammation was responsible for endothelial dysfunction and impaired nitric oxide availability independent of lipid profile alterations [111]. Moreover, McCoy et al. [62] found that thyroxine supplementation was significantly associated with CVD, which supports the fact that the administration of this medication does not decrease the occurrence of this outcome. Autoimmunity itself may be an independent risk factor for CVD.

As both diseases increase inflammatory parameters and cytokines and cause endothelial dysfunction, a relationship between polyautoimmunity (RA and AITD) and the occurrence of CVD is not surprising.

Although antimalarial use was not significant in the bivariate analysis, we decided to keep the variable in the multivariate analysis. This is because this medication has been associated with a better cardiovascular outcome, improved glycated hemoglobin in patients with type 2 diabetes mellitus [112], enhanced glycemic control in patients with RA and SLE, and a reduced risk of developing diabetes mellitus in those patients [113, 114] in several reports. Furthermore, these medications influence cardiovascular risk by lowering total cholesterol levels [115, 116], which strengthens the hypothesis that reducing inflammation is important in reducing the risk of CVD in RA patients. This seemed to be the case with our RA patients with AITD. 

It is noteworthy that most of the retrieved articles were from Europe followed by North American countries such as United States and Canada. This could be linked to the theory that Hashimoto's thyroiditis is the most frequent cause of spontaneously acquired hypothyroidism in industrialized countries. Few developing countries have data on AITD prevalence. These are Egypt, Iran, and Morocco. The latter reports the smallest prevalence of what we found in our literature search. 

Considering thyroid antibodies, the prevalence is also heterogeneous. It is widely accepted that among these thyroid antibodies the most frequent is TPOAb compared to TgAb [6]. This has happened in almost all the studies that reported data on both antibodies [57, 70, 77, 80, 82, 87], and in our cohort. Nonetheless, this is not the case in the article from Japan by Nakamura et al. [79] in which they found the same prevalence for both antibodies. In addition, two studies from Egypt, one by El-Sherif et al. [74] and the other by Assal et al. [73], found an increased prevalence of TgAb, respectively. However, the study by Mousa et al. [57] found a higher prevalence for TPOAb in Egypt. A small sample size in these situations may be the best explanation for these contradictory findings. In Latin America, Rivero et al. [75], in an Argentinean setting, found a prevalence of 20% for TgAb while Gonçalves et al. [77] in Brazil found a prevalence of 15% for TPOAb and 7% for TgAb. Ruggeri et al. [81] demonstrated an increasing prevalence of THAb with time; pathologies different from AITD (RA and SS) exhibit increasing prevalences as well. It is noteworthy that this study also demonstrates that beyond an association of RA with Hashimoto's thyroiditis, antibodies to thyroid hormones (i.e., T3 and T4) may also foster the development of hypothyroidism. 

Nevertheless, as the first study in Latin America that describes the relationship, our results do not differ from what has been reported in other latitudes. We report a prevalence of 9.8% of AITD in RA subjects, a TPOAb prevalence of 37.78%, and a TgAb prevalence of 20%. Although a prevalence of TgAb that was similar to Rivero's was found, the antibody prevalences in this study differ from those mentioned earlier by Gonçalves et al. [77]. Almost two times more of each antibody was found in our study than what they reported. In addition the AITD prevalence in RA patients is higher than in the general population from Latin America. According to Marsiglia the prevalence of AITD in the general population in Venezuela is 4.2% [117].

 With respect to RA severity we only found one abstract that assessed the link between AITD and RA. Charles et al. [118] did not found a relationship between the presence of thyroid antibodies and the occurrence of anti-CCP although they did with PTPN22R620W allele. Likewise, in our cohort we did not find a correlation between AITD and proxy variables for RA severity such as erosions, biologic agent use, the presence of anti-CCP [119], and EAMs (data not shown). The reason why this association between AITD and RA severity has not been studied is not immediately apparent. One cannot but hypothesize that many of these studies are cross-sectional in nature and because the importance of DAS28 and HAQ is along a timeline, it is not relevant to include these variables in the analysis. 

**Figure 2:**
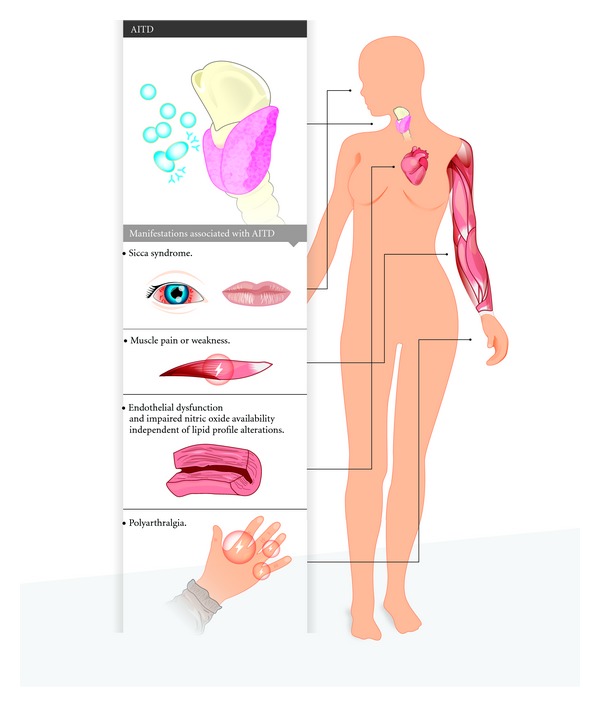
AITD manifestations in patients with RA. AITD manifestations may resemble those presented by RA. Some symptoms are exacerbated when both diseases co-occur. See text for details.

In nonautoimmune hypothyroidism however, Cojocaru-Gofita et al. [120] found that women with AR and clinical hypothyroidism had a higher DAS28 score compared to RA women without clinical hypothyroidism. Kang et al. [121] found that in Korean patients with AR subclinical or clinical hypothyroidism was associated to the occurrence of positive titers of anti-CCP. Also, Delamere et al. [122] found that thyroid dysfunction is associated with increased mean duration and incidence of morning stiffness. It is important to consider these reports because some of these patients may have AITD and this could be related to the severity of RA.  Figure 2 illustrates the main symptoms in patients with AITD and AR.

The importance of this Colombian cohort is worth considering. We attempt to add further knowledge with respect to the characteristics of RA in minorities in Latin America, a region about which literature on this topic is scarce. 

We are aware of our study limitations. First of all, information bias could be present in our analysis as not all patients with RA were systematically evaluated for all the variables. This is the case for thyroid antibodies, which were only assessed in patients that had some type of thyroid disturbance. This included 135 patients for TPOAb and 125 for TgAb. Secondly, the cross-sectional nature of the study does not allow us to infer causality. Another limitation is one that is linked to all searches—some articles may have escaped our search and, thus, some regions may have been overlooked. Additionally, the articles found had small sample sizes. It is important to consider the heterogeneity in the definition of AITD as well. In contrast, our strengths are our number of participants, a well-described cohort of RA patients, and the multicenter validation of RA cases. To our knowledge, this is the first paper that addresses this particular topic from a global perspective.

There were more patients with TPOAb and TgAb than with a clinical diagnosis of AITD. Linked with the idea that autoantibodies are predictors of disease [123, 124], it is important to remain vigilant in following the clinical course of these patients; TPOAb and TgAb are known to predict AITD. This was demonstrated in the Wickham cohort [97]. Patients within accepted TSH reference range and having the aforementioned antibodies had a greater risk of developing overt hypothyroidism (i.e., AITD). Also TPOAb has been shown to predict development of AITD in pregnant women [125]. A careful assessment of those patients with a normal range of TSH but presenting specific antibodies should be done.

To conclude, we have found that AITD is not uncommon in RA patients. The range has its lower limit in 0.5% and it goes up to 27%. For TgAb, this prevalence ranges from 6% to 31% and for TPOAb, also from 5% to 37%. The prevalence of AITD and antibodies in our cohort falls within these ranges. Our literature search indicates that literature is scarce and, therefore, more research is needed on this topic, particularly in developing countries. The findings in this study justify a prospective analysis that follows RA patients diagnosed with AITD. They also support routine screening for CVD among these patients. These results may help to further study the common mechanisms of autoimmune diseases, to improve patients' outcome, and to define public health policies. An international consensus to accurately diagnose AITD is warranted.

## Figures and Tables

**Figure 1:**
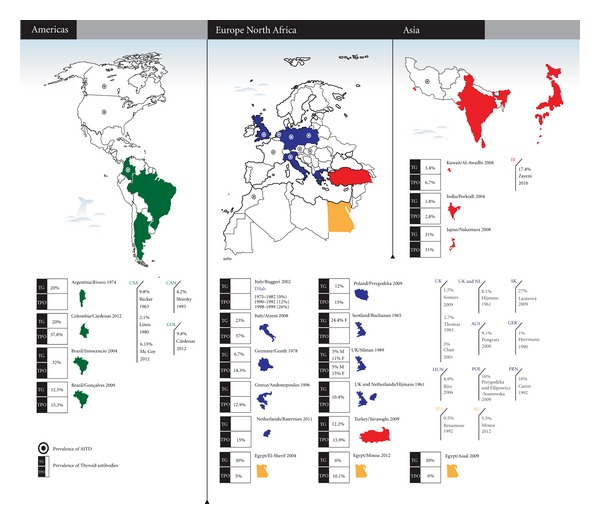
Prevalence of AITD and antibodies worldwide.

**Table 1:** Characteristics of 800 patients with RA.

Characteristic	
Age (years)	51.92 (12.19)^a^
Age at onset (years)	39.58 (12.35)^a^
RA duration (years)	10 (14)^b^
Educational level (years)	11 (9)^b^
Body mass index	24 (5.8)^b^
DAS28	3.63 (2.12)^b^
HAQ	1.05 (1.31)^b^

Sociodemographic	*n*/*N* (%)

Female	650/800 (81.3)
Low educational level	264/692 (38.2)
Low socioeconomic status	234/780 (30.0)
Current smoking	85/768 (11.1)
Household duties	254/684 (37.1)

Clinical aspects	

Type 2 diabetes	32/737 (4.3)
Dyslipidemia	184/752 (24.5)
Hypertension	208/752 (27.7)
Thrombosis	39/738 (5.3)
Cardiovascular disease	173/781 (22.2)
Body mass index > 25	394/681 (57.9)
Abdominal obesity	460/683 (67.4)
Aspirin use	105/653 (16.1)
Abnormal cholesterol	179/333 (53.8)

RA characteristics	

Disease duration > 10 years	393/703 (55.9)
Erosions	349/451 (77.4)
EAMs with CVD	402/793 (50.7)
Rheumatoid factor+	573/717 (79.9)
Anti CCP+	312/384 (81.3)
Methotrexate	702/794 (88.4)
DMARD (any)	783/794 (98.6)
Antimalarials	633/793 (79.8)
Steroids	705/793 (88.9)
Biological Agents	276/794 (34.8)

Autoimmunity	

Autoimmune thyroid disease	78/800 (9.8)
Systemic lupus erythematosus	11/709 (1.6)
Sjögren's syndrome	24/800 (3.0)
Polyautoimmunity	113/800 (14.1)
Polyautoimmunity^c^	35/800 (4.8)
MAS	17/714 (2.4)
Familial autoimmunity FDR	104/800 (13.0)
ANAs+	310/448 (69.2)
Anti Ro+	43/287 (15.0)
Anti La+	20/285 (7.0)
TPOAb+	51/135 (37.8)
TgAb+	26/125 (20.8)

ANAs: antinuclear antibodies; CCP: cyclic citrullinated peptide; CRP: C-reactive protein; CVD: cardiovascular disease; DAS28: disease activity score; DMARD: disease modifying antirheumatic drugs; EAM: extra-articular manifestations; ESR: erythrocyte sedimentation rate; FDR: first degree relatives; HAQ: health assessment questionnaire; MAS: multiple autoimmune syndrome; RA: rheumatoid arthritis; TgAb: anti-thyroglobulin; TPOAb: anti-thyroperoxidase enzyme.

^a^ Mean (standard deviation).

^b^ Median (interquartile range).

^c^ Without taking AITD into account.

**Table 2:** Bivariate analysis of categorical variables.

Characteristic	RA with AITD	RA without AITD	OR	95% CI	*P *
MAS	10/61 (16.39)	7/650 (1.08)	18.01	6.57–49.30	<0.0001
Type 2 Diabetes	12/72 (16.67)	20/665 (3.01)	6.45	3.00–13.83	0.008
Methotrexate	75/78 (96.15)	627/716 (87.57)	3.54	1.09–11.49	0.024
Female	72/78 (92.31)	575/719 (79.97)	3.01	1.2–7.05	0.008
Thrombosis	9/71 (12.68)	30/667 (4.50)	3.01	1.4–6.78	0.003
Anti La+	6/39 (15.38)	14/246 (5.69)	3.01	1.08–8.3	0.04*
Anti Ro+	11/39 (28.21)	32/248 (12.90)	2.65	1.20–5.84	0.013
Abnormal BMI	37/69 (53.60)	250/612 (40.8)	1.67	1.01–2.76	0.042
Low educational level	15/59 (25.4)	249/633 (39.3)	0.52	0.28–0.96	0.035
Abnormal cholesterol	17/43 (39.53)	162/290 (55.86)	0.51	0.26–0.99	0.045
RF+	50/73 (68.49)	523/644 (81.21)	0.50	0.29–0.85	0.01
Polyautoimmunity	78/78 (100)	35/722 (4.8)	N/A	N/A	N/A
TPOAb+	51/54 (94.44)	0/81 (0.00)	N/A	N/A	N/A
TgAb+	26/50 (52.00)	0/75 (0.00)	N/A	N/A	N/A

*Fisher's exact test.

95% CI: 95% confidence interval; AITD: autoimmune thyroid disease; BMI: body mass index; N/A: not assessed; OR: odds ratio; RA: rheumatoid arthritis; RF: rheumatoid factor; TgAb: anti-thyroglobulin; TPOAb: anti-thyroperoxidase enzyme.

**Table 3:** Bivariate analysis of continuous variables.

Characteristic	RA with AITD	RA without AITD	*P *
	Mean ± SD	Mean ± SD	
Age	52.26 ± 12.39	51.88 ± 12.24	0.029
	**Median ± IQR**	**Median ± IQR**	
Educational level (*y*)	14 ± 7	11 ± 9	0.006
Body mass index	25.5 ± 6.3	23.9 ± 5.9	0.006

AITD: autoimmune thyroid disease; IQR: interquartile range; RA: rheumatoid arthritis; SD: standard deviation.

**Table 4:** Associated factors with AITD in RA (multivariate analyses).

Characteristic	*B *	AOR	95% CI	*P *
Thrombosis	3.19	24.41	2.73–218.43	0.004
Diabetes	2.61	13.61	1.61–114.96	0.016
BMI > 25	1.44	4.22	1.19–14.93	0.025
Rheumatoid factor+	0.95	2.58	0.33–19.88	0.36
Methotrexate use	0.90	2.48	0.27–22.36	0.418
Female	0.46	1.58	0.34–7.42	0.56
Abnormal cholesterol	−1.22	0.29	0.08–1.10	0.069
Duration disease > 10 years	−1.32	0.27	0.07–1.05	0.058
Low educational level	−1.82	0.16	0.03–0.88	0.036
Antimalarials	−2.29	0.10	0.02–0.57	0.01

95% CI: 95% confidence interval; AOR: adjusted odds ratio; BMI: body mass index.

**Table 5:** Prevalence of AITD diagnosis in RA patients.

Author publication date	Location	Study population	Diagnostic criteria of RA	Diagnostic criteria of AITD	Number of Cases	Frequency	Prevalence %
Africa							
Mousa et al. 2012 [57]	Egypt	F: 80% A: 36.3	ACR 1987	Lab.	217	12	5.5
Benamour et al. 1992 [58]	Morocco	F: 87.4% A: 34	ARA	N/A	404	2	0.5
America							
Cárdenas et al. 2012*	Colombia	F: 81.3% A: 51.92	ACR 1987	Lab.	800	78	9.8
Shiroky et al. 1993 [59]	Canada	F: 76. A: 58.7	ARA	Biopsy	119	7	4.2
Becker et al. 1963 [60]	USA	N/A	ARA	Histology	51	5	9.8
Linos et al. 1980 [61]	USA	F: 74.1% A: N/A	ARA	N/A	521	11	2.1
McCoy et al. 2012 [62]	USA	F: 69% A: 55.8 ± 15.7	ACR	Lab.	650	40	6.1
Europe							
Hijmans et al. 1961 [23]	Europe^#^	N/A	ARA 1959	N/A	86	7	8.1
Pongratz et al. 2000 [63]	Austria	F: 88.3% A: N/A	ARA	N/A	383	35	9.1
Caron et al. 1992 [64]	France	N/A	N/A	N/A	131	21	16
Herrmann et al. 1990 [65]	Germany	F: 86% A: N/A	N/A	US, Lab.	201	2	1
Biro et al. 2006 [66]	Hungary	N/A	ARA	Lab.	185	9	4.9
Somers et al. 2009 [67]	UK	F: 92% A: N/A	GPRD	GPRD	22888	337	1.5
Thomas et al. 1983 [68]	UK	F: N/A A: 52	N/A	NR	295	8	2.7
Chan et al. 2001 [69]	UK	F: 90% A: N/A	ARA	Lab.	64	2	3.0
Przygodzka and Filipowicz-Sosnowska 2009 [70]	Poland	F: 100% A: 56 ± 13	ACR	Lab.	100	16	16.0
Lazúrová et al. 2009 [71]	Slovakia	F: N/A A: 52.2 ± 2	N/A	US, Lab.	68	19	27.0
Middle East							
Zayeni et al. 2010 [72]	Iran	F: 87.1% A: 49.05	N/A	Lab. Clinical examination	224	39	17.4

^#^Location not stated. Collaboration between the UK and The Netherlands.

*Current series.

N/A: Not available; F: Proportion of females; A: Age at time of assessment (standard deviation); ARA-ACR: RA diagnostic criteria 1987; UK: United Kingdom, US: Ultrasound, USA: United States of America, GPRD: General Practice Research Database; Lab.: Laboratory criteria.

**Table 6:** Prevalence of TPOAb and TgAb in RA patients.

Author publication date	Location	Study population	Number of Cases	Diagnostic	Frequency		Prevalence %	
				criteria of RA	TPOAb	TgAb	TPOAb	TgAb
Africa								
Assal et al. 2009 [73]	Egypt	F: 66.6% A: 26.8	30	ACR 1987	2	9	6.0	30.0
El-Sherif et al. 2004 [74]	Egypt	N/A	20	N/A	N/A	N/A	5.0	30.0
Mousa et al. 2012 [57]	Egypt	F: 80% A: 36.3	217	ACR 1987	22	13	10.1	6.0
America								
Cárdenas et al. 2012*	Colombia	F: 81.3% A: 51.92	125–135**	ACR 1987	51	26	37.8	20.8
Rivero et al. 1974 [75]	Argentina	N/A	50	N/A	N/A	10	N/A	20.0
Innocencio et al. 2004 [76]	Brazil	N/A	25	ACR 1987	8		32.00	
Gonçalves et al. 2009 [77]	Brazil	F: 86% A: 50 ± 10	72	ACR 1987	11	9	15.3	12.5
Asia								
Porkodi et al. 2004 [78]	India	N/A	N/A	N/A	21	13	2.8	1.8
Nakamura et al. 2008 [79]	Japan	F: 82.76% A: 61 ± 14	29	N/A	9	9	31.0	31.0
Europe								
Hijmans et al. 1961 [23]	Europe^#^	N/A	86	ARA 1959	9		10.4	
Atzeni et al. 2008 [80]	Italy	F: 81% A: 47 ± 16	70	ACR 1987	26	16	37.0	23.0
Ruggeri et al. 2002 [81]^*≠*^	Italy	N/A	N/A	N/A	N/A	N/A	1975–1982: 0	
							1990–1992: 12	
							1998-1999: 26	
Genth et al. 1978 [82]	Germany	N/A	105	N/A	15	7	14.3	6.7
Andonopoulos et al. 1996 [83]	Greece	N/A	101	N/A	N/A	N/A	12.9	N/A
Raterman and Nurmohamed 2012 [84]	NL	N/A	N/A	ACR 1987	N/A	N/A	15	N/A
Magnus et al. 1995 [85]	Norway	N/A	100	N/A	N/A		^§^	
Przygodzka and Filipowicz-Sosnowska 2009 [70]	Poland	F: 100% A: 56 ± 13	100	ACR 1987	15	12	15.0	12.0
Buchanan 1965 [86]	Scotland	F: 100%	N/A	N/A	N/A	N/A	N/A	24.4
Yavasoglu et al. 2009 [87]	Turkey	F: 82%	82	ARA 1959	13	10	15.9	12.3
Silman et al. 1989 [88]	UK	N/A	N/A	N/A	N/A	N/A	males: 5 females: 15	males: 5 females: 11
Middle East								
Al-Awadhi et al. 2008 [89]	Kuwait	F: 79.1% A: 38.3	177	ACR 1987	12	6	6.7	3.4

N/A: Not available; F: Proportion of females; A: Age at time of assessment (standard deviation); NL: The Netherlands; UK: United Kingdom; USA: United States of America.

*Current series.

**See text for details.

^#^Location not stated. Collaboration among UK and NL.

^§^Compared to the prevalence in the normal population, patients with rheumatoid arthritis have a higher prevalence of both antibodies.

^*≠*^Prevalence assessed in three time points. Only valid for thyroid hormone antibodies (THAb).
